# Evaluating pre- and post-operation plasma miRNAs of papillary thyroid carcinoma (PTC) patients in comparison to benign nodules

**DOI:** 10.1186/s12885-019-5849-0

**Published:** 2019-07-15

**Authors:** Mahsa Rezaei, Amir Mahdi Khamaneh, Nosratollah Zarghami, Armin Vosoughi, Shahriar Hashemzadeh

**Affiliations:** 1 Department of General Surgery, Tabriz University of Medical Sciences, Imam Reza Hospital, Golgasht Street, Tabriz, Iran; 2 Department of Molecular Medicine Faculty of Advanced Medical Sciences, Tabriz University of Medical Sciences, Tabriz, Iran; 3 Department of Clinical Biochemistry and Laboratory Medicine, Faculty of Medicine, Tabriz University of Medical Sciences, Tabriz, Iran; 4 Neurosciences Research Center (NSRC), Faculty of Medicine, Tabriz University of Medical Sciences, Imam Reza Hospital, Golgasht Street, Tabriz, Iran; 5 Tuberculosis and Lung Disease Research Center, Tabriz University of Medical Sciences, Tabriz, Iran

**Keywords:** Benign nodules, MicroRNA, Papillary thyroid carcinoma, Plasma samples

## Abstract

**Background:**

Thyroid cancer is the most common endocrinology cancer that its incidence has increased in recent decades. miRNAs are new biomarkers in recent studies in the diagnosis and follow-up of these patients.

**Methods:**

Blood and thyroid tissue samples were obtained from two groups of included patients (PTC and benign nodules), pre- and post-operation. miRNAs were extracted from these plasma samples and were measured quantitatively. After cDNA synthesis, qPCR was carried out. Then tissue samples were investigated, and their relation to miR expression was studied. These results were analyzed by paired- and independent samples t-test, and non-parametric tests.

**Results:**

miR-222 and miR-181a declined in PTC patients before and after surgery, significantly (*P* < 0.001 for both groups), with no significant difference in control group before and after surgery (*P* = 0.61 for miR-222 and *P* = 0.06 for miR-181a). The difference between the two groups, pre-and post-operation, was statistically significant (*P* = 0.01 for miR-222 and *P* < 0.001 for miR-181a). Comparing case and control groups, pre- and post-operatively, yielded no significant difference, in miR-155-5p levels (*P* = 0.61 and *P* = 0.53, respectively). Comparing PTC and control groups before surgery showed a significant difference (*P* = 0.01), while no significant difference was observed comparing them after surgery, in miR146-a (*P* = 0.27). Our results depicted a higher miR-155-5p and miR-146a expression before surgery than after it (*P* < 0.001 in both groups, for both miRs). We found a significant relationship between miR-222 and BRAFV600E mutation and significantly higher levels of miR-181a with increasing tumor size in PTC patients.

**Conclusion:**

miR-222 showed overexpression in all PTC cases, which is indicative of a relation between miRNA and PTC. Also, comparing miR-181 and miR-146a showed a significant difference between cancerous and benign cases. miR-155-5p as an inflammatory factor, showed no significant changes, comparing two groups.

## Background

Thyroid cancer is the most common malignancy amongst the endocrine cancers, and its incidence has increased recently, especially in case of papillary thyroid cancer (PTC), and is still rising, mostly due to increased diagnosis [1]. This growth in diagnosis might be related to diagnostic imaging utilities, as well as fine-needle aspiration (FNA) biopsy. However, this increase has partly led to overdiagnosis and overtreatment [2, 3].

In a report by Lim et al. [4], the incidence of thyroid cancer was reported to increase by 3%, each year. The collected data from their study revealed a 211% incline in thyroid cancer incidence, with PTC being the most incident and less aggressive one, from 1974 to 2013. On the other hand, the estimated new cases of PTC in 2017 in the United States has been up to 56′870 cases, while, 2010 deaths have been roughly estimated to take place in a year [5].

PTC tends to be the most prevalent, yet one of the most curable malignancies of thyroid [6]. Though many proto-oncogenes have been involved in the pathogenesis of PTC (e.g., RET, NTRK1, RAS, and BRAF) [7], still a lot is to be discovered about the pathogenesis of the disease. One fact is that not only the mutations in coding genes are involved in disease development, but also genetic variations in non-coding regions have been reported to play a part in PTC. Of these non-coding genes, microRNA (miR) genes have gained attention and have been shown to play distinct roles in the process of the disease [8].

Although a lot is to be discovered regarding miRs functions, they tend to act as negative regulators of gene expression, which by binding to 3′-untranslated region of mRNAs (messenger RNA) display variable effects on gene expression level, hence altering target protein levels [9]. Meanwhile, deregulation or overexpression of miRs has been reported in different types of cancers. For instance, prooncogenic miRs (e.g., miR-155 and miR-21) have been depicted to be over-expressed in some cancers, whereas, tumor suppressors (miR-15a and miR-16) have been down-regulated in malignancies [8].

Likewise, in PTC, different results have been reported regarding the alterations of miRs in comparison to normal states. There have been some studies about the up-regulation of some miRs such as miR-146, miR-221, miR-222, miR-155, miR-181 and miR-34 [8–10]. Whereas, miRs like miR-138 and miR-98 have been reportedly down-regulated in PTC [9].

Nonetheless, current diagnostic methods for differentiating malignant lesions from benign nodules are restricted to FNA and surgical pathological examination. Since, FNA is not determinant, with low predictive value in cases of suspicious malignancy, atypia of undetermined significance and follicular neoplasms, an unnecessary lobectomy or thyroidectomy might be performed, to diagnose properly [11]. Thus, more precise and less invasive methods should be developed, to gain a more reliable and feasible way of diagnosis. Based on many studies, miRs could be a potential hotspot for researches to reach such a level.

Despite evaluating different miRs in PTC, a lot is undiscovered and needs to be shed light upon. Meanwhile, the target genes of these miRs are partly known for us, along with the fact that clinicopathologic features of the disease have not been related to miR expression changes. In this study, we evaluated the miRs that have been shown to be overexpressed in PTC in some studies (i.e., miR-222 and miR-181), next to the miRs that have not been elucidated clearly in PTC (i.e., miR155–5p and miR-146a). In the meantime, we assessed the alterations in these miRs in comparison to non-PTC thyroid lesions. Finally, we evaluated the relation between the clinicopathologic findings of the PTC patients and their miR status were evaluated.

## Methods

### Data collection

This study was conducted between March 2017 to July 2018, in Imam Reza Hospital, Tabriz, Iran. The patients had already undergone FNA, due to thyroid nodules, and the ones with the diagnosis of PTC and benign nodules were included. Amongst the patients in endocrinology outpatient clinic of Imam Reza hospital, 38 cases were diagnosed within the case recruitment period, 8 of which were excluded. Thirty patients were enrolled as the control group with benign nodules.

All patients were given information about the study, and they all filled out informed consent forms. This study was confirmed by the ethics committee of Tabriz University of Medical Sciences. The tenets of the Declaration of Helsinki were followed.

### Patient and samples information

Two groups of patients were evaluated in case and control groups, regarding the plasma levels of miR-222, miR-155-5p, miR-146a and miR-181a, and tissue levels of BRAF, both before and after thyroidectomy. Case group was comprised of 30 patients that were diagnosed as PTC by FNA, while the control group included 30 patients with benign thyroid nodules. All the demographic data including age, sex and the pathologic characteristics of the tumor are brought in Table 1.

All the patients that had the history of comorbid disorders and previous history of malignancy such as colorectal cancer and melanoma were excluded from the study. Also, patients with any contraindications for FNA were not included in the study. On the other hand, a family history of thyroid cancers was another exclusion criterion for our study.

### Samples preparation

5 cc of blood were obtained from all patients, before surgery. A couple of drops were spilled out intentionally to prevent an infestation of the samples with skin cells and germs, and the rest of the samples were collected in ethylenediamine tetraacetic acid (EDTA) tubes. Then, the blood was centrifuged at 3000 RPMI for 5 min. The sterile plasma was then collected and moved into DNase and RNase free tubes. The collected plasma was stored at − 70 °C refrigerator, until RNA extraction. The same procedure was also carried out for samples that were collected 2–6 weeks after surgery. For blinding the experiment, all samples were evaluated altogether, to prevent any biases by the lab analysts.

### RNA extraction

Total RNA was extracted by Trizol reagent (Ambion life technologies, UK). Main components of this reagent include guanidinium thiocyanate and phenol that ultimately the denature proteins and prevent the undesired effects of RNase on RNA content. Our procedures followed the exact procedures of the company for total RNA extraction from liquid samples, except for increasing centrifuge time (up to 13,000 g for 30 min), replacing ethanol with isopropyl alcohol and storing the extracts in − 20 °C for one night, after adding ethanol, just to have the small RNA deposit.

RNA concentration (ng/ul) was measured with Nano-Drop device (Spectrophotometer 2000/2000c), and the quality of RNA was estimated based on 230/260 and 260/280 data, where the first one depicts the mineral contamination and the second shows the protein contamination. The ideal data for both is 2, while for the TRIzol method, 1.60 and 1.80 are acceptable amounts for plasma samples.

### cDNA synthesis

Our assay included two steps of cDNA (complementary DNA) synthesis and qPCR (quantitative polymerase chain reaction) to determine the quantity of housekeeping and target miRNAs in specimens. For cDNA synthesis, as well as PCR amplification, we used Biomir High Sensitivity MicroRNA Kit (ZistRoyesh.co), which exploits stem-loop construction for each miRNA and housekeeping miRNA. The end product of this process for each miRNA includes a hairpin structure that contains targeted miRNA in 5̛ and a complementary DNA for this target in 3̛ end of the complete hairpin.

### RT-PCR

The mentioned end products were considered as patterns for the RT-PCR. The levels of miRNA expression were measured by quantitative RT-PCR, by the above kit, which contains a specific primer for each miRNA and general primer for part of stem-loop, the product has sufficient length to be detectable by RT-PCT. Whereas, amplification consists of denaturation step: 20 s on 95° and annealing-extension step: 60 s on 60°, for 35–40 cycles and final melt analysis step between 55°-95°, along with initial 15 min in 95° to activate Hot StarTaq Master Mix kit.

The main goal of normalization in RT-PCR is to create a reference point, in order to refer all the measurements to this reference point. Normalization process was established by housekeeping miRNAs and efficiency index of PCR reaction, that was assessed during the amplification experiments. RT-PCR data was quantified relatively based on comparing the target miR Ct (cycling time) values with housekeeping miR. Relative quantification depends on the comparison of the target and reference genes that are mainly showed by fold changes in miRs values. Presence of cDNA in each assay mirrored miRNAs content in the sample and displayed by Ct value in Real-Time PCR device. Ct value was determined as the points where the PCR curve meets the threshold level. The mathematical model that is widely applied for relative quantification approach is the ΔΔCt model. The assay series contain Ct data for control samples and Ct data for case samples. For each sample, target gene and housekeeping gene have been measured. Serially diluted aliquots in 10 fold formats apply for each PCR run to evaluate efficiency rate of each assay series.

The input amount of cDNA is presented as Ct, by altering the curve format to a logarithmic setting (which is available in the settings of the device). ∆Ct for each gene is then calculated by subtracting the Ct number of target sample from that of the control sample.

### BRAFV600E sampling

Tissue samples were washed with normal saline and stored in-70 °C. To extract DNA, tissue was harvested and after vaporization of the fluid nitrogen, on over 300 mg of the tissue, DNA extraction by Nucleospin tissue kit (by MN GmbH), according to the kit procedure was, carried out. DNA concentration (ng/ul) was measured with Nano-Drop device (Spectrophotometer 2000/2000c), and the quality of DNA was estimated based on 230/260 and 260/280 data, where the first one depicts the mineral contamination and the second shows the protein contamination. The acceptable amount of extracted DNA for the kit was 1.8 to 2. The BRAFV600E mutation was measured by BRAF MutlD kit (produced by Amitis Co), which is a qualitative mutation test kit for amplification of the nucleic acids based on real-time PCR.

### Statistical analysis

To analyze the clinical data, independent samples t-test was used. Statistical significance was set at *P* ≤ 0.05. Non-parametric tests were used in order to depict the relation of clinical and pathologic findings with miR expression levels. Also, to depict the demographic data, descriptive analysis was carried out.

## Results

### General findings

Mean ± standard deviation (SD) for age in the case group (PTC patients), was 44.76 ± 11.11, while in the control group (benign nodules) was 43.36 ± 8.82. There was no significant difference between groups (*P* = 0.12).

In case group there were 11 males (36.7%) and 19 females (63.3%), while in the control group 17 patients (56.7%) were males, and 13 patients (43.3%) were females. This difference between the two groups was not statistically significant (*P* = 0.09).

On the other hand, we also recorded the clinical and pathologic characteristics of the participants, which are depicted in details in Table 1. Nonetheless, among the case group patients, BRAFV600E mutation was present in 13 cases (43.3%), while in the control group there was solely one positive case (3.3%) for this mutation. The difference between the two groups was statistically significant (*P* < 0.001).

**Table 1:** Demographic findings and tumor characteristics in case and control groups

			Frequency	Percent	*P*-value
Age at surgery (years)	Case group	Below 45	13	43.3%	**0.12**
		Above 45	17	56.7%	
	Control group	Below 45	17	56.7%	
		Above 45	13	43.3%	
Sex	Case group	Female	19	63.3%	**0.09**
		Male	11	36.7%	
	Control group	Female	13	43.3%	
		Male	17	56.7%	
BRAFV600E mutation	Case group	Positive	13	43.3	**< 0.001**
		Negative	17	56.7	
	Control group	Positive	1	3.3	
		Negative	29	96.7	
Involved lobe	Case group	Right	14	46.7	**0.67**
		Left	12	40	
		Both	4	13.3	
	Control group	Right	16	53.3	
		Left	12	40	
		Both	2	6.7	
Tumor/nodule size	Case group	Below 2 cm	14	46.7	**0.39**
		Above 2 cm	16	53.3	
	Control group	Below 2 cm	12	40	
		Above 2 cm	18	60	
Capsular involvement	Case group	Yes	4	13.3	**0.50**
		No	26	86.7	
	Control group	Yes	5	16.7	
		No	25	83.3	
Lymph node involvement	Case group	Yes	10	33.3	**< 0.001**
		No	20	66.7	
	Control group	Yes	0	0	
		NO	30	100	
Lymphadenectomy	Case group	Yes	9	30	**0.001**
		No	21	70	
	Control group	Yes	0	0	
		No	30	100	

### Main findings

**Fig. 1:**
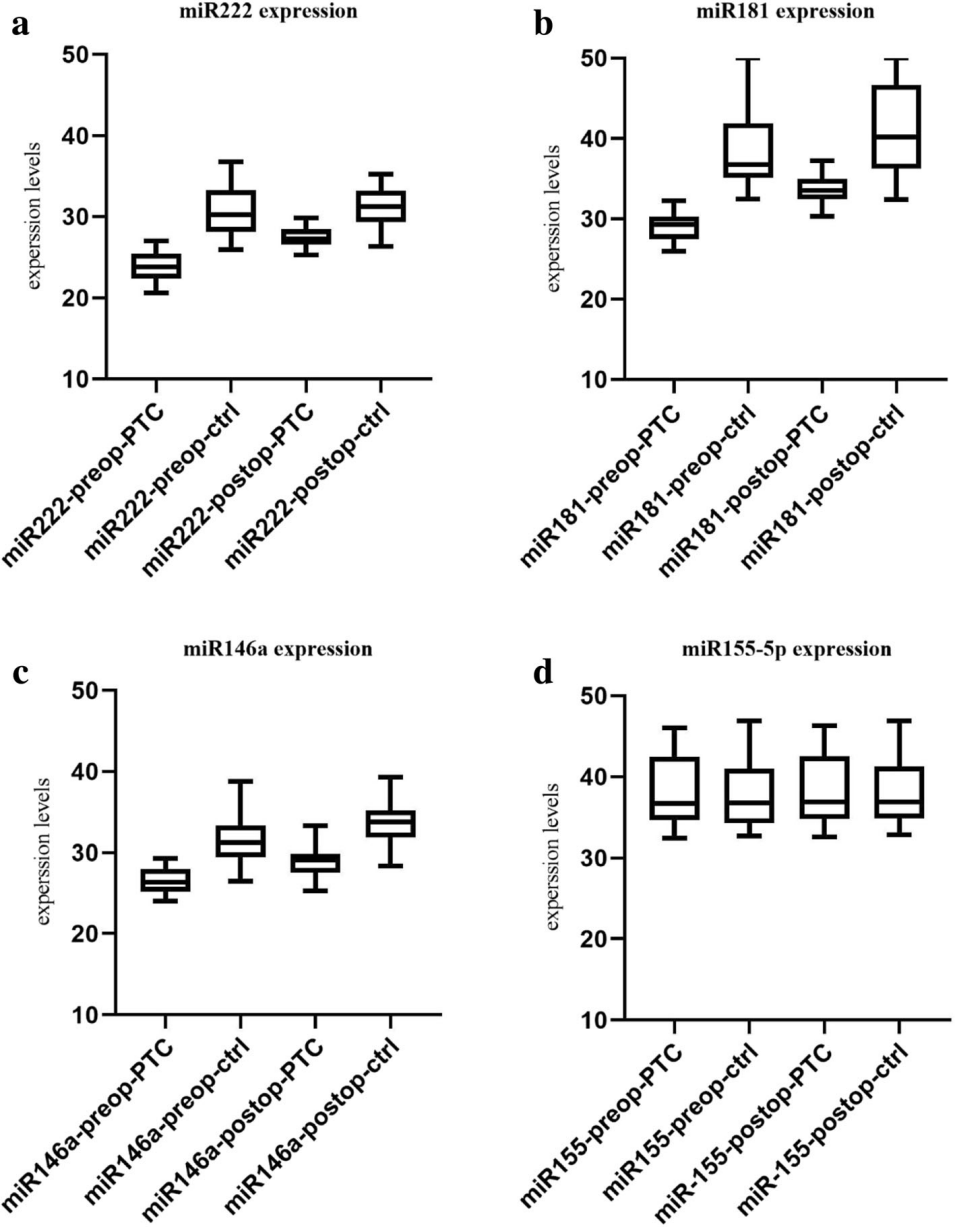
Boxplots of all evaluated miRs, in PTC and benign groups, pre- and post-operation, **a**. miR-222 expression, **b**. miR-181a expression, **c**. miR-146a expression, and **d**. miR-155-5p expression

We evaluated miR-222, miR-181a, miR-155-5p and miR-146a before and after surgery in PTC and benign nodules. The details regarding the Pre-operation (pre-op) and post-operation (post-op) miRNAs are brought in Tables 2 and 3. The expression levels of the miRs are brought in Fig 1 a-d.

**Table 2:** Selected miRNAs and their alterations pre- and post-operation in case and control groups

			Median	Mean ± SD	*P*-value
miR-222	PTC	Pre-op	23.82	23.85 ± 1.88	< 0.001
		Post-op	27.28	27.47 ± 1.67	
	Control	Pre-op	30.28	30.66 ± 2.85	0.61
		Post-op	31.25	31.04 ± 2.51	
miR-181a	PTC	Pre-op	29.27	29.06 ± 1.70	< 0.001
		Post-op	33.53	33.60 ± 1.75	
	Control	Pre-op	36.79	38.57 ± 4.97	0.06
		Post-op	40.20	40.99 ± 5.64	
miR-155-5p	PTC	Pre-op	36.69	38.29 ± 4.37	< 0.001
		Post-op	36.91	38.49 ± 4.48	
	Control	Pre-op	36.78	37.93 ± 4.30	< 0.001
		Post-op	36.93	38.18 ± 4.28	
miR-146a	PTC	Pre-op	26.36	26.53 ± 1.61	< 0.001
		Post-op	29.13	28.89 ± 1.82	
	Control	Pre-op	31.25	31.41 ± 2.80	< 0.001
		Post-op	33.75	33.52 ± 2.39	

The data regarding miRs are presented here both by median and mean±SD, while the P-values depict the difference of distinct miRs within PTC or control groups (P-values were determined by independent samples t-test)

**Table 3:** Comparing the difference in miR expression, in case and control groups, before and after surgery

		*P*-value
miR-222	Before surgery	0.01
	After surgery	< 0.001
miR-181a	Before surgery	< 0.001
	After surgery	< 0.001
miR-155-5p	Before surgery	0.61
	After surgery	0.53
miR-146a	Before surgery	0.01
	After surgery	0.27

#### miR-222

Evaluating miR-222 depicted that this miRNA is roughly 6.6 fold higher in the pre-operation state than post-operation. This difference in PTC patients before and after surgery was statistically significant (*P* < 0.001), while there was no significant difference in the control group before and after surgery (*P* = 0.61). The difference between case and control groups before and after thyroidectomy was significant, statistically (*P* = 0.01 and *P* < 0.001, respectively).

#### miR-181a

Our findings regarding miR-181a indicated that its pre-operation level is almost 2.2 folds higher than post-operation levels. Subsequently, the difference between pre- and post-operation expression in PTC patients was statistically significant (*P* < 0.001), whereas this difference in case group was not significant (*P* = 0.06). On the other hand, the difference between case and control groups, before and after operation, was statistically significant (*P* < 0.001 for both).

#### miR-155-5p

Studying the differences in miR-155-5p showed a 1.06-fold change, depicting a higher miRNA expression before surgery than after it. The confirmatory analysis showed a significant decline of expression in PTC patients, comparing their miRNA status before and after surgery, as well as control group patients (*P* < 0.001 for both). However, comparing case and control groups, pre- and post-operation, yielded no significant difference, statistically (*P* = 0.61 and *P* = 0.53, respectively).

#### miR-146a

a 1.38-fold increase was observed in pre-operation levels, in comparison to post-operation results. Nonetheless, both case and control groups showed a significant decline in their miR-146a expression, after surgery (*P* < 0.001). Though, comparing PTC and control groups before surgery showed a significant difference between two groups (*P* = 0.01), no significant difference was observed comparing them after surgery (*P* = 0.27).

The data regarding miRs are presented here both by median and mean ± SD, while the *P*-values depict the difference of distinct miRs within PTC or control groups (*P*-values were determined by independent samples t-test)

#### miRNA relation with clinicopathological findings

Evaluating miRs relation with clinical and pathological findings, showed that miR-222 has a significantly higher expression in BRAFV600E-positive PTC patients (*P* < 0.001). In the meantime, in PTC patients, both before and after surgery, a significant relationship between miR-146a and BRAFV600E mutation was observed, meaning that the positive BRAF mutation, caused elevated levels of miR-146a. Meanwhile, miR-222 and miR-181a depicted significant higher levels of expression in female PTC patients (*P* = 0.03 and *P* = 0.006, respectively).

However, only in miR-181a was a significant relation with size, in a manner that higher size was significantly related to higher miR-181a expression in PTC patients (*P* = 0.04). Finally, no significant relation was observed between evaluated miRs and lymph node involvement, capsular involvement and the involved lobe of thyroid. The details are brought in Table 4.

**Table 4:** Relation between miRs and demographic, clinical and pathologic findings in both groups

		miR-222		miR-181a		miR-155-5-P		miR-146a	
		Pre-op	Post-op	Pre-op	Post-op	Pre-op	Post-op	Pre-op	Post-op
Age	Case	0.95	0.96	0.20	0.46	0.32	0.28	0.41	0.60
	Control	0.67	0.10	0.01	0.05	0.60	0.67	0.43	0.41
Lymphadenectomy	Case	0.25	0.10	0.42	0.46	0.30	0.24	0.28	0.14
	Control								
Lymph node involvement	Case	0.24	0.31	0.59	0.28	0.50	0.45	0.32	0.09
	Control								
Sex	Case	*0.03*	0.46	*0.006*	0.12	0.42	0.50	0.20	0.16
	Control	0.18	0.41	0.08	0.05	0.39	0.32	0.27	0.49
Size	Case	0.95		*0.04*		0.38		0.78	
	Control	0.32		0.65		0.13		0.14	
BRAFV600E	Case	*< 0.001*	0.98	0.60	0.64	0.30	0.30	*< 0.001*	*< 0.001*
	Control	0.22	0.86	0.60	0.52	0.32	0.32	0.18	0.18
Capsular involvement	Case	0.15	0.66	0.09	0.83	0.11	0.11	0.71	0.80
	Control	0.27	0.13	0.26	0.24	0.59	0.52	0.04	*0.01*
Involved lobe of thyroid	Case	0.66	0.28	0.71	0.36	0.47	0.47	0.57	0.62
	Control	0.72	0.45	0.87	*0.02*	0.43	0.40	0.96	0.87

All *P*-values were obtained by Mann-Whitney U test, whereas the significant findings are *italic*

## Discussion

Previous studies have evaluated miRs as prognostic and diagnostic factors for malignancy [12, 13]. In this study, we evaluated the expression levels of miR-222, miR-181a, miR-146a and miR-155-5p in plasma of PTC patients, and compared it to patients with benign thyroid nodules. Furthermore, we compared pre-operation levels of the mentioned miRs to their post-operation levels. Our results depicted significantly higher levels of miR-222, miR-181a, and miR-146a, in case group patients than the control group, before surgery. However, no significant difference was observed in miR-155-5p comparing PTC and control patients, before surgery. Meanwhile, our results were indicative of a significant overexpression of miR-222 and miR-181a, after surgery, in PTC patients. Whereas, no significant difference was shown evaluating miR-146a and miR-155-5p.

On the other hand, our results regarding the PTC patients, before surgery, depicted significantly higher levels of all measured miRs (i.e., miR-222, miR-181a, miR-146a, and miR-155-5p) than post-operation expression status. However, in case group patients, per se, only miR-146a and miR-155-5p levels declined significantly after surgery, while this significant difference was not observed in miR-222 and miR-181a levels, comparing pre- and post-operation levels.

Our study revealed a significant relationship between miR-222 and BRAFV600E mutation in PTC patients. On the other hand, we acquired significant higher levels of miR-181a with tumor size in PTC patients.

miR-222 has been shown to be an ever-overexpressed miR in PTC cases, as it might be the signature of PTC [10]. It also has been shown that miR-222 rises in all races in PTC [9], introducing this miR as a potential diagnostic tool for PTC cases. Similar to many studies [11, 14, 15], our study showed a substantial elevation of miR-222 in PTC patients, which insists on its role in the pathology of thyroid malignancy. This miRNA is mentioned to be targeting *p27*, *p57*, and *PUMA* [11–16], playing a role as an oncogene. Also, miR-222 overexpression has been associated with reduction in KIT proteins [15, 17]. Regardless, evaluating PTC tissue samples yielded overexpressed levels in many studies [18, 19]. Meanwhile, this overexpression in some cases has been attributed to increased aggression in PTC cases. Protein phosphatase 2 regulatory subunit B alpha (PPP2R2A) has been articulated as another target for miR-222, leading to increased invasion, in these cases. Also, AKT signaling pathway has also been targeted by these miRs, causing more aggressive behavior of the tumor [18]. All these, introduce miR-222 as a potential diagnostic method, as well as a target for treatment plans.

In another study [20], the levels of miR-222 and miR-146b were shown to be significantly higher in the serum of recurrent PTC patients, in comparison to patients without recurrent PTC. They reported a 10.8-fold increase in miR-222 levels, which was similar to ours (6.6-fold increase in PTC patients in comparison to benign nodules), as well as an 8.9-fold increase in miR-146b.

However, different studies have utilized various methodologies, either from the aspect of samples or from aspect of methods, for investigating miRNA expression [21]. For instance, in a study by He et al. [15], PTC tissue samples were investigated for miRNA levels. Whereas, in a study by Lee et al. [20], serum levels of miRs were evaluated. Their results depicted an overexpression of miR-146b, miR-221, and miR-222, in PTC patients, compared with healthy normal cases. Our study, too, evaluated the plasma samples of PTC patients, whereas our control group was benign nodules, which indicates a specific role for miR-222 overexpression in PTC. Another study, confirmed the above mentioned findings (i.e., overexpressed miR-222, miR-146b along with miR-221) in PTC tissue samples [22].

In a similar study by Yu et al. in 2012 [11], circulating miRs were measured in PTC patients, in comparison to benign and healthy controls. Similar to ours, their results depicted an increase in miR-222, miR-151-5p, and let-7e in serum samples. This study suggested that these molecular biomarkers might possess potential specificity and sensitivity to be as a surrogate method for differentiating PTC from benign nodules, thus introducing these miRs valuable for the diagnosis of PTC. Unlike our study, this study reported a significantly elevated level of miR-222 in lymph node-positive patients. Their findings depicted a significant increase in miR-151-5p level with tumor size growth, whereas our findings showed the same significant elevation in miR-181a levels.

Lee et al., conducted a similar study in 2015 [23], evaluating the levels of miR-146b and miR-155, in the serum of PTC patients. The results were similar to ours, showing a significant overexpression of miR-155, along with miR-146b in PTC. They also reported a role for these miRs in discriminating PTC and benign nodules. Also they showed miR-155 could be beneficial in differentiating benign cases from PTC in the presence of lymph node metastasis. Unlike our study, this study showed no significant overexpression in miR-222, as well as miR-221. However, they showed a significant relation between miR-222, miR-155, and miR-146b with tumor size, while our study showed no relation. Yet, miR-181a was the only measured miRNA in our study that depicted a significant relation with tumor size. Another study [24], evaluated the miR-155 levels in PTC tissue, showed an overexpression of miR-155 in comparison to adjacent tissues, which was not in accordance with our study. However, they reported a significant relationship between this overexpression with increased tumor size, extrathyroid invasion, lymph and node metastasis. Our study, did not find any significant relation between the expression of miR-155-5p and aggressiveness of the tumor. This might be due to the racial differences between two studies.

Our study indicated a significant rise in miR-146a in PTC patients. This miRNA has been shown to be involved in tumor suppression and immune responses (i.e., Toll-like receptor and cytokine signaling) and is being regulated by NF-kappa B (NF-κB) [25]. Subsequently, miR-146a tends to act as a feedback inhibitor in NF-κB signaling pathway and inflammation, where it has been suggested to play a linking role between inflammation and cancer [26]. Though, limited recent studies have evaluated this miRNA in PTC [27], a study by Jazdzewski et al. [28], reported a genetic predisposition to PTC, in patients with a polymorphism in pre-miR-146a, leading to an elevation in miR-146a mature levels. They showed a 1.4- to 2.7-fold increase in miR-146a in PTC tissue samples using TaqMan stem-loop RT-PCR, while our results showed a roughly 1.36-fold increase in miR-146a expression in PTC patients plasma samples, using stem-loop RT-PCR. Altogether, this finding might be indicative of a possible role of the NF-κB pathway in thyroid tumorigenesis. Nonetheless, Graham et al. [29], reported a significant decrease in miR-146a in serum of PTC patients in comparison to benign thyroid nodules. One reason for this difference, is their small sample size.

In another study [30], the relation between miRs and expression of Thyroid Hormone Receptor β (THRB) was evaluated in PTC patients. This study revealed that higher levels of miR-221, miR-146a, and miR-21 caused a decline in expression of THRB, leading to reduced tumor suppressor role of this gene. However, this study failed to show any relations between miR-181a and THRB expression, in PTC patients. This shows that miR-181a is either innocent or not playing a role in THRB pathway, in PTC pathophysiology.

A study by Le et al. in 2017 [31], evaluated the role of miR-181a, in PTC tissue samples. The results of this study were similar to ours, as it depicted a significant elevation in these patients. They suggested that overexpression of miR-181a might play an oncogenic role by targeting RB1, leading to accelerated cell cycle progression and reduced apoptosis, among cancerous cells.

Circulating miRs have been indicated to be stable, reportedly [29–32]. This might be an important point, since the stability of these miRs could introduce them as diagnostic tools, in many malignancies. Subsequently, our findings and evaluated studies here, depict the significant alterations of multiple miRs (esp. miR-222 and miR-181a) in PTC, expressing them as a potential diagnostic method.

## Conclusion

Our results depicted significant over-expressed levels of plasma miR-222, miR-181a, and miR-146a in PTC patients compared to benign nodules, while miR-155-5p showed no significant difference between case and control groups. High levels of miR-146a, and miR-155-5p in some of the control group patients, could be attributed to the inflammatory causes, which after surgery had declined significantly. Our findings could suggest a potential role for miR-222, miR-181a, and miR-146a in differentiating PTC cases from benign nodules. Taken together, these findings reveal the importance of miR alterations in PTC, which might value them as less invasive diagnostic tools. Our findings could be an important update for coming up with novel diagnostic for easier detection of PTC. Further studies, with larger sample size, could focus on the same miRs along with other mentioned miRs, in order to confirm or disapprove our findings in population along with determining the diagnostic cut-off for them.

## Data Availability

The datasets used and/or analyzed during the current study are available from the corresponding author on reasonable request.

## Abbreviations

cDNA: Complementary DNA
ct: Cycling time
FNA: Fine needle aspiration
miR & miRNA: Micro RNA
PTC: Papillary thyroid carcinoma
qPCR: Quantitative polymerase chain reaction
RT-PCR: Reverse transcription polymerase chain reaction
